# Reproduction, infection and killer-cell immunoglobulin-like receptor haplotype evolution

**DOI:** 10.1007/s00251-016-0935-9

**Published:** 2016-08-12

**Authors:** Bridget S. Penman, Ashley Moffett, Olympe Chazara, Sunetra Gupta, Peter Parham

**Affiliations:** 1 Department of Zoology, University of Oxford, Tinbergen Building, South Parks Road, Oxford, OX13PS UK; 2 Department of Pathology, University of Cambridge, Cambridge, CB2 1QP UK; 3 Department of Structural Biology, Stanford University, Stanford, CA 94035 USA; 4 Department of Microbiology and Immunology, Stanford University, Stanford, CA 94035 USA

**Keywords:** Killer-cell immunoglobulin-like receptors (KIRs), Natural killer cells, Infectious disease, Reproduction, Human evolution

## Abstract

Killer-cell immunoglobulin-like receptors (KIRs) are encoded by one of the most polymorphic families in the human genome. KIRs are expressed on natural killer (NK) cells, which have dual roles: (1) in fighting infection and (2) in reproduction, regulating hemochorial placentation. Uniquely among primates, human *KIR* genes are arranged into two haplotypic combinations: *KIR A* and *KIR B*. It has been proposed that *KIR A* is specialized to fight infection, whilst *KIR B* evolved to help ensure successful reproduction. Here we demonstrate that a combination of infectious disease selection and reproductive selection can drive the evolution of *KIR B*-like haplotypes from a *KIR A*-like founder haplotype. Continued selection to survive and to reproduce maintains a balance between *KIR A* and *KIR B*.

**Electronic supplementary material:**

The online version of this article (doi:10.1007/s00251-016-0935-9) contains supplementary material, which is available to authorized users.

## Introduction

Killer-cell immunoglobulin-like receptors (KIRs) are expressed on the surface of natural killer cells (NK cells)—the major effector lymphocytes of innate immunity. Specific *KIR* genotypes, genes or alleles are associated with differential responses to a variety of infectious diseases (Gao et al. 2010; Hirayasu et al. 2012; Khakoo et al. 2004; Knapp et al. 2010; Martin et al. 2002, 2007; Seich al Basatena et al. 2011). Furthermore, in addition to their role in fighting infection, NK cells are involved in pregnancy. Uterine NK cells (uNK cells) regulate how fetal placental cells remodel the spiral arteries that supply nutrients and oxygen to the developing feto-placental unit. In humans, maternal *KIR* genotype has been shown to affect the likelihood of severe pregnancy syndromes (Hiby et al. 2004, 2008, 2010; Nakimuli et al. 2015), and birth weight itself (Hiby et al. 2014).

KIRs can be activating or inhibitory. Multiple *KIR* genes are found in a 150-kb cluster on chromosome 19. Strikingly, all human populations—even those which have experienced extreme bottlenecks (Gendzekhadze et al. 2006)—possess two *KIR* haplotypes with distinctly different gene contents. The *KIR A* haplotype has largely fixed gene content, with mostly genes encoding inhibitory KIRs; the *KIR B* haplotype has a more variable gene content and contains several genes encoding activating KIRs. Other primate species display a high degree of KIR haplotypic diversity, but no equivalent organisation into *KIR A* and *KIR B*-like entities (de Groot et al. 2015; Guethlein et al. 2015). Why *KIRs* should have been segregated in this way in humans, and why *KIR A* and *KIR B* are always maintained in every human population, is an evolutionary phenomenon demanding explanation.

One hypothesis that has been proposed is that *KIR A* haplotypes are specialized to ensure success in fighting infection, and *KIR B* haplotypes are specialized to ensure success in reproduction (Parham 2005, 2008; Parham and Moffett 2013). This is in keeping with the observation that *KIR A* homozygous individuals exhibit better clearance of hepatitis C infection (Khakoo et al. 2004), but *KIR B* homozygous mothers are protected against the potentially fatal pregnancy syndrome pre-eclampsia (Hiby et al. 2004, 2010; Nakimuli et al. 2015). However, population genetic frameworks within which to test whether a combination of such selective pressures can indeed promote the evolution of *KIR A* and *KIR B* haplotypes have so far been lacking. Here, we integrate links between *KIR* genotype, infectious disease and reproduction into a single model. We demonstrate that a combination of infectious disease selection and reproductive selection can drive the evolution of both *KIR A*—like and *KIR B*-like haplotypes from a *KIR A*-like ancestor.

## Methods

**Fig. 1:**
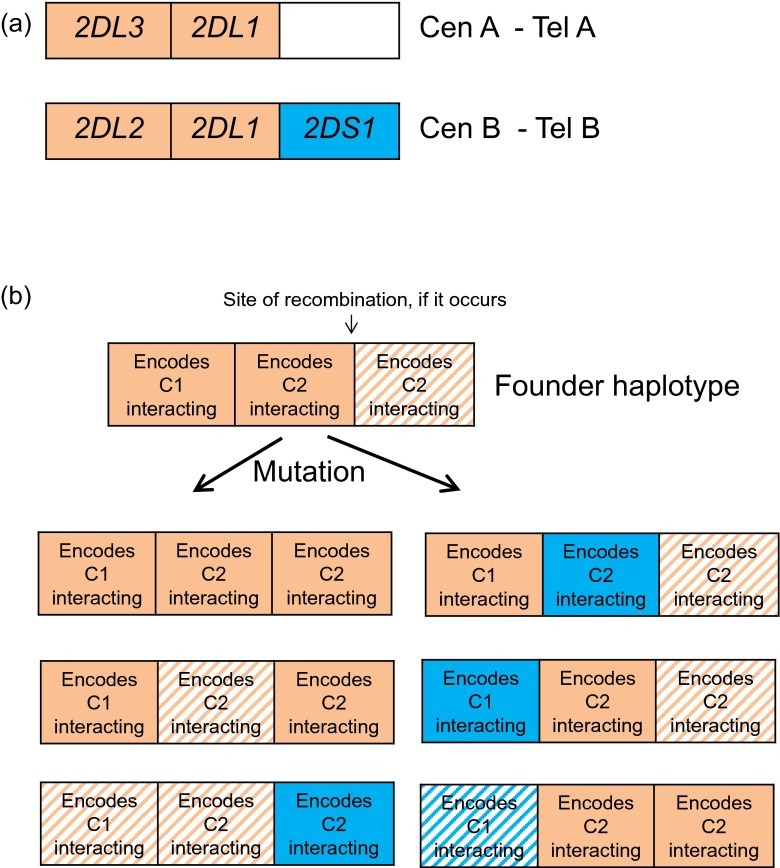
**Hypothetical** ***KIR*** **haplotypes that can compete within the model.** As described in the text, we considered haplotypes consisting of three linked *KIR* genes. One encodes a KIR that can bind C1 (and is thus equivalent to human *KIR2DL2* or *KIR2DL3*) and the other two encode KIRs that can bind C2 [equivalent to *KIR2DL1* or *KIR2DS1*, and perhaps also KIR2DS5*006 (Nakimuli et al. 2015)]. For simplicity, these specificities were not allowed to mutate. Panel **a** shows how the most frequent *KIR A* and *B* haplotypes observed in Caucasians would appear within our framework. Panel **b** illustrates the founder haplotype used in the model and a non-exhaustive range of possible haplotypes that could arise through mutation within the model. Genes encoding inhibitory KIRs are indicated in orange, genes encoding activating KIRs in blue. Functional (expressed) genes are indicated by solid colours; non-functional (pseudogene) genes are indicated by hashed colours. When recombination was allowed to take place, it took place only between the second and third loci in the cluster. This reflects the situation in humans where recombination seems to occur most frequently between the centromeric region of the *KIR* cluster (which may contain genes encoding C1 or C2 interacting KIRs) and the telomeric region of the *KIR* cluster (which may contain a gene encoding a C2 interacting KIR).

Many KIRs have been shown to bind directly to class I major histocompatibility complex (MHC) molecules expressed on the surface of other cells. In humans, MHC molecules are known as human leukocyte antigens, or HLAs. For simplicity, we will henceforth consider only KIR/HLA interactions involving HLA-C, the dominant HLA ligand for KIRs which is present in all individuals. HLA-C molecules form two types of KIR ligand, depending on the amino acid residue at position 80. These are known as C1 and C2, and a balance between *HLA-C* alleles encoding C1 or C2 ligands is observed in all human populations. In order to explore the generation of A and B *KIR* haplotypes, we simulated a haplotype containing three possible *KIR* genes (Fig. 1). One gene encodes a KIR that can bind C1 (and is thus equivalent to human *KIR2DL2* or *KIR2DL3*) and two genes encode KIRs that can bind C2 [equivalent to *KIR2DL1* or *KIR2DS1*, and perhaps also KIR2DS5*006 (Nakimuli et al. 2015)]. Mutation rates were incorporated in the model such that (1) genes could switch between encoding activating or inhibitory KIRs; (2) genes could switch between being expressed or pseudogenes, and (3) the strength (magnitude) of the inhibitory or activating signal associated with the encoded KIR could change. In this way, a range of possible three-gene *KIR* haplotypes could be generated at random within each simulation, of varying degrees of similarity to those seen in human populations.

Diploid combinations of *KIR* haplotypes and *HLA* genotypes (*HLA*-*C1* homozygous; *HLA-C2* homozygous and *HLA*-*C1*/*C2* heterozygous) were used to define individuals in an individual-based model. Every generation, all individuals had to survive infectious disease challenge, where their probability of success was linked to their *KIR/HLA* genotype (further details in the following sections). Survivors were then randomly sampled with replacement to be the parents of the next generation, such that a maximum of *K* pregnancies took place. The success of each simulated pregnancy was determined by the combination of maternal *KIR* and fetal *HLA* genotype (further details in the following sections), and only surviving offspring could contribute to the next generation. The individual-based process of first surviving infection and then reproducing was iterated over *T* generations in each simulation. With each birth, came fixed probabilities of the aforementioned KIR mutations or recombination (see Table 1).

**Table 1:** Parameter definitions, and how probabilities of events within the model were calculated

Item	Parameter or calculation
Number of generations in simulation	*T*
Maximum number of births per generation	*K*
Probability of an individual dying from infection with a pathogen for which detecting missing self is important.	*θ* for all individuals who are not able to express both C1 and C2 and at least one functional inhibitory KIR recognising each, otherwise 0.
Probability of an individual dying from infection with a pathogen for which NK cell activation state is important.	$\mu \left(1-{A}_{x_L\le x\le {x}_H}\right)$ where *μ* = the maximum possible mortality rate and ${A}_{x_L<x<{x}_H}$ = the proportion of the individual’s NK cell response that falls within the optimum zone for surviving infection.
Probability of a pregnant female and her fetus being lost from the population due to excessively inhibited uNK cells	$p.{A}_{y\le {y}_T}$ where *p* = the maximum risk and ${A}_{y\le {y}_T}$ = the proportion of the uterine NK cell response equal to or below the critical value *y*_*T*_.
Probability of a pregnant female and her fetus being lost from the population due to excessively activated uNK cells	$q.{A}_{y_U\le y}$ where *q* = the maximum risk and ${A}_{y_U\le y}$ = the proportion of the uterine NK cell response equal to or above the critical value *y*_*U*_.
Per birth probability of a mutation affecting the magnitude of the signal transmitted by the receptor encoded by a *KIR* gene to NK cells in the presence of its ligand.	*m*_*1*_ If a mutation occurred, a value drawn from a uniform distribution between −1 and +1 was added to the existing signal magnitude assigned to that particular KIR in that individual’s genome. If the resulting magnitude was <0.1, a magnitude of 0.1 was assigned.
Per birth probability of a mutation in the pseudogene versus expressed status of any *KIR* gene.	*m*_*2*_ For simplicity there was no difference in the rate at which genes defined as pseudogenes became expressed, or genes defined as expressed became pseudogenes: this was a symmetrical mutational process.
Per birth probability of a mutation in the activating or inhibitory status of the receptor encoded by any *KIR* gene.	*m*_*3*_ For simplicity there was no difference in the rate at which genes defined as encoding activating receptors switched to encode inhibitory receptors, or genes defined as encoding inhibitory receptors switched to encode activating receptors: this was a symmetrical mutational process.
Rate at which recombination occurs between the second and third *KIR* genes in the haplotype.	*r*

### Infectious disease selection

We included two types of infectious disease selection in our simulations:

1. *Selection from HLA-evading pathogens*. Individuals heterozygous for *HLA-C1* and *HLA-C2* whose genome encoded at least one expressed inhibitory KIR specific to each of C1 and C2 were assumed to have an advantage against pathogens which exploit particular forms of HLA-evasion. This advantage is captured by parameter *θ* (see Table 1).
2. *Selection from pathogens for which the degree to which NK cells are activated or inhibited is important*. We generated, for each individual in the model, a normal distribution of possible NK cell activation states derived from their *KIR/HLA* genotype. In this way, the various KIR/HLA interactions possible for a specific individual (i.e. the different types and magnitudes of signals associated with those interactions) could combine to generate a single phenotype (see Electronic supplementary material (ESM) Methods for further details We defined a range of optimum NK cell activation states to survive infection (*x*_L_ < *x* < *x*_H_). The per generation probability of a given individual dying from this type of infection was then given by $\upmu \left(1-{A}_{x_L\le x\le {x}_H}\right)$, where ${A}_{x_L\le x\le {x}_H}$ is the proportion of that individual’s NK cell distribution that fell within the optimum range.

The biological justifications for these assumptions were as follows:

1. Certain viruses down-regulate MHC molecules and/or express decoy MHC molecules as a means of immune evasion. In humans, inhibitory KIRs help NK cells to combat such viruses by detecting a lack of HLA (‘missing self’). When HLAs are present, inhibitory KIRs for which those HLAs are ligands prevent NK cell killing. If such inhibitory signals are lost, the NK cell kills the abnormal cell. In keeping with evolutionary simulations that suggest the presence of multiple possible inhibitory KIR-HLA ligand pairs is advantageous against a cytomegalovirus-like pathogen (Carrillo-Bustamante et al. 2014), we have assumed that HLA *C1/C2* heterozygotes who express inhibitory KIRs that can recognise both C1 and C2, and thus have the maximum possible number of KIR-HLA ligand pairs protected by a missing-self mechanism in our model, are protected against extra mortality from pathogens capable of this or similar forms of HLA-evasion.
2. KIRs differ in the relative inhibitory or activating effects they have on NK cells (Moesta et al. 2008), and the strength of this inhibition or activation seems to affect the possibility of mortality. For both hepatitis C and hepatitis B, the genotype associated with the weakest possible inhibitory interaction involving HLA-C (homozygosity for *KIR2DL3* and *HLA-C1*) offers an increased chance of clearing the infection (Gao et al. 2010; Khakoo et al. 2004; Knapp et al. 2010). Conversely, genotypes which encode certain highly inhibitory KIRs and their known HLA ligands are associated with delayed progression to AIDS (Martin et al. 2007). Activating KIRs are also directly implicated in improved responses: the activating receptor KIR3DS1 together with its putative ligand HLA-Bw4 is associated with a delayed progression to AIDS (Martin et al. 2002) and reduced susceptibility to opportunistic infections in individuals with HIV (Qi et al. 2006).

### Reproductive selection

We generated, for every pregnancy that occurred within each simulation, a normal distribution of uterine NK (uNK) cell activation states, based on the inhibition/activation signal strengths of the KIRs encoded in the mother’s genome that could interact with the HLA-C supplied by the father (see ESM Methods for full details). Both excessive inhibition of uNK cells and excessive activation of uNK cells can be risky. The probability of the failure of a pregnancy due to excessive inhibition of uNK cells was $p.{A}_{y\le {y}_T}$ where *p* = the maximum mortality rate and ${A}_{y\le {y}_T}$ = the proportion of the uNK cell response equal to or below the critical value *y*_*T*_. The probability of the failure of a pregnancy due to excessive activation of uNK cells was $q.{A}_{y_U\le y}$ where *q* = the maximum risk and ${A}_{y_U\le y}$ = the proportion of the uNK cell response equal to or above the critical value *y*_*U*_. In both cases, we assumed that the failure of a pregnancy led to the deaths of both mother and infant.

The biological justifications for these assumptions were as follows:

- uNK cells associate closely with the developing trophoblast (Moffett-King 2002). KIRs expressed on uNK cells bind to HLA-C molecules expressed by invading trophoblast cells, and the interaction between fetal *HLA-C* and maternal *KIR* genotype has been shown to influence pregnancy outcome (reviewed in Moffett and Colucci 2015). Excessive inhibition of uNK cells is a risk factor for pregnancy disorders where placentation is poor, e.g. pre-eclampsia, fetal growth restriction and recurrent miscarriage (Hiby et al. 2004, 2008, 2010, 2014; Nakimuli et al. 2015). At the other extreme, greater activation of uNK cells is positively correlated with higher birth weights (Hiby et al. 2014). Paternally derived C2 expressed by the trophoblast seems to be particularly associated with these effects, hence our use of paternal HLA-C only to define such risks in the model. It seems that the degree of activation or inhibition of uNK cells upon their interaction with fetal trophoblast cells has a direct effect on the effectiveness of placentation; most likely on the level of blood supplied through remodelled spiral arteries in the uterine wall (reviewed in Moffett and Colucci 2015).
- The importance of these observations becomes apparent when we consider the obstetric dilemma: an evolutionary challenge unique to modern humans. Bipedalism places physical restrictions on the structure of the pelvis, but encephalization (increasing brain size) demands that babies be born with heads as large as possible. Childbirth for *Homo sapiens* is therefore an exceptionally dangerous process for both mother and baby, owing to the extremely tight fit of the head of the fetus through the birth canal (Wittman and Wall 2007). Given that, as we have just discussed, the greater the level of activation of uNK cells, the greater the birth weight, the obstetric dilemma is likely to generate intense selective pressures on KIRs.
- In our model, we assume that both excessive inhibition and excessive activation of uNK cells are capable of killing women and fetuses during pregnancy. Pre-eclampsia, which is clearly linked to uNK cell inhibition, can lead to fatal eclampsia. Obstructed labour, particularly common in humans for the reasons just outlined, is associated with higher birth weights and therefore likely to be linked to excessive activation of uNK cells. Obstructed labour, too, can kill both mother and fetus, or can leave the mother with major injuries, hence our assumption that both extremes of uNK cell inhibition and activation have the potential to be extremely disadvantageous.

### Choice of parameter values

For computational feasibility we set the maximum number of births per generation (i.e. the maximum population size), *K* = 1000. In terms of the probability of a mutation arising in the population at all, a mutation rate of 1 × 10^−5^ in a population of 1000 individuals is equivalent to a mutation rate of 1 × 10^−7^ in a population of 100,000 individuals. Given the relatively small population size in our simulations, for the scenarios presented in the main text we have used relatively high mutation rates—which made it possible for a wider range of potential *KIR* haplotypes to emerge and compete with one another. In the ESM Results, we show that our overall results do not depend on the specific mutation or recombination rates applied, provided rates are not so low that no mutations can emerge in our simulated populations (Fig. S1).

Parameters *p, q, μ* and *θ* generate different types and strengths of selective pressure. As such, the absolute values of each parameter are less important than their relative values. We seek to define which selective pressures are vital in order to obtain a balance between *KIR-A* and *KIR-B*, not to claim that specific values for each parameter have applied throughout human history.

*p* is the maximum per-pregnancy probability of pregnancy failure and maternal death associated with insufficiently activated uNK cells. Present-day rates of pre-eclampsia, a proxy which will substantially underestimate parameter *p* because *p* only applies to the most at risk genotypes, are 6.2 to 10.2 % in American populations (Nakimuli et al. 2014). *q* is the maximum per-pregnancy probability of pregnancy failure and maternal death associated with excessively activated uNK cells. Present-day rates of obstructed labour, a proxy which will likewise underestimate parameter *q*, are between 3 and 6 % (Dolea and AbouZahr 2003). Given the uncertainty in the historical effects of pre-eclampsia and obstructed labour on human survival, and to make sure that we explored a wide range of possibilities, in our sensitivity analyses we tested values of *p* and *q* between 0 and 0.4 (see ESM Results and Fig. S2).

*θ* is the selection pressure from pathogens for which having two types of HLA-C KIR ligand, both protected by a missing-self mechanism, is an advantage. As a shorthand, we refer to these as HLA-evading pathogens. *μ* is the selection pressure from pathogens for which the degree to which NK cells are activated or inhibited affects the survival of the host. In each generation of the model, *θ* and *μ* define the maximum probability of dying from either type of infectious disease before reproducing, i.e. the probability of dying for the most at risk genotypes. Importantly, these parameters do not represent the case fatality rates of specific pathogens: rather the overall probability of dying from a particular sort of infectious disease during childhood. We know from human history that specific pathogens come and go. Smallpox, for example, is a relatively recent burden that was only a significant cause of mortality within the last several hundred generations; *falciparum* malaria, similarly, seems to only have emerged within the last ten thousand years. We can, however, be confident that infectious diseases have plagued modern humans for as long as we have existed. Parameters *θ* and *μ* allow our model to ask which types of relationship between infectious diseases, KIRs and HLAs (if any) are most likely to generate a balance between KIR A and KIR B haplotypes. Just as for parameters *p* and *q*, in our sensitivity analyses we allowed parameters *θ* and *μ* to take values between 0 and 0.4, to capture a wide range of possible selection scenarios.

## Results

### Selection from HLA-evading pathogens alongside reproductive selection is essential to generate *A* and *B* like *KIR* haplotypes

**Fig. 2:**
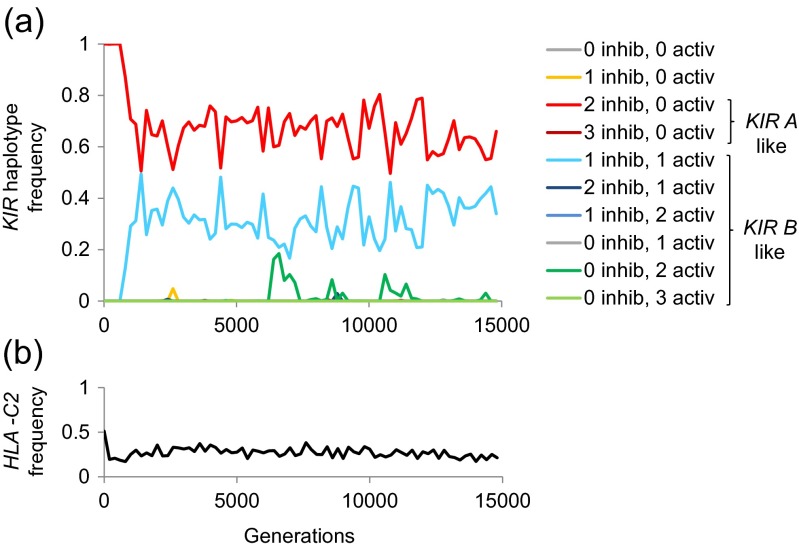
**The emergence of** ***KIR B*** **alongside a founder** ***KIR A*** **haplotype. **Panel **a** illustrates the frequencies of different possible *KIR* haplotypes as they evolve in a single simulation. Each *KIR* haplotype is defined by its number of functional genes encoding inhibitory (‘inhib’) or activating (‘activ’) KIRs. Mutations also occurred in the strength of activation or inhibition associated with different allelotypes, but it is not practical to represent all these in this figure. In the figure legend, we identify subsets of *KIR A*-like and *KIR B*-like haplotypes, where *KIR A*-like haplotypes encode at least two inhibitory KIRs and no activating KIRs, and *KIR B*-like haplotypes encode at least one activating KIR. Panel **b** illustrates the frequency of *HLA-C2* in the same population. Parameter values were as follows: *T* = 15,000; *K* = 1000; *θ* = 0.15; *μ* = 0.2; *p* = 0.26; *q* = 0.1; *m1* = 0.001; *m2* = 1 × 10^−5^; *m3* = 1 × 10^−5^ and *r* = 0.01. The founder haplotype encoded a functional C1 interacting inhibitory KIR with a signal magnitude of 1.5 (see ESM Methods for further information on how inhibitory or activating signals with different magnitudes generated by different KIRs were incorporated in the model); a functional C2 interacting inhibitory KIR with a signal magnitude of 5, and a pseudogene C2 interacting inhibitory KIR with a signal magnitude of 5. The signal magnitudes for the functional C1 and C2 interacting KIRs in the founder KIR A haplotype were chosen to reflect the observation that, in present-day populations, KIR2DL3 + C1 has a weaker inhibitory effect than KIR2DL1 + C2. However, all KIR signal magnitudes were free to mutate at rate *m1* (see Table 1 for further details of mutational processes). Threshold values for the ranges of NK cell activation states that responded best to different challenges (see Methods) were: *x*_*L*_ = −6, *x*_*H*_ = −5, *y*_*T*_ = −2 and *y*_*U*_ = 1.

Our closest evolutionary relatives, chimpanzees, possess both C1 and C2, but lack an obvious *KIR A/B* haplotypic structure. Human *KIR A* haplotypes have more in common with chimpanzee *KIR* haplotypes than human *KIR B*. We thus began each simulation with balanced frequencies of C1 and C2 encoding *HLA* alleles and the population fixed for a *KIR A*-like haplotype (Fig. 1). We sought to identify the conditions under which a *KIR B*-like haplotype emerged alongside the evolving *KIR A* haplotype, and the two were still maintained together at the end of a 15,000 generation simulation. Figure 2 illustrates the time course of one of our simulations displaying this behaviour of interest.

**Fig. 3:**
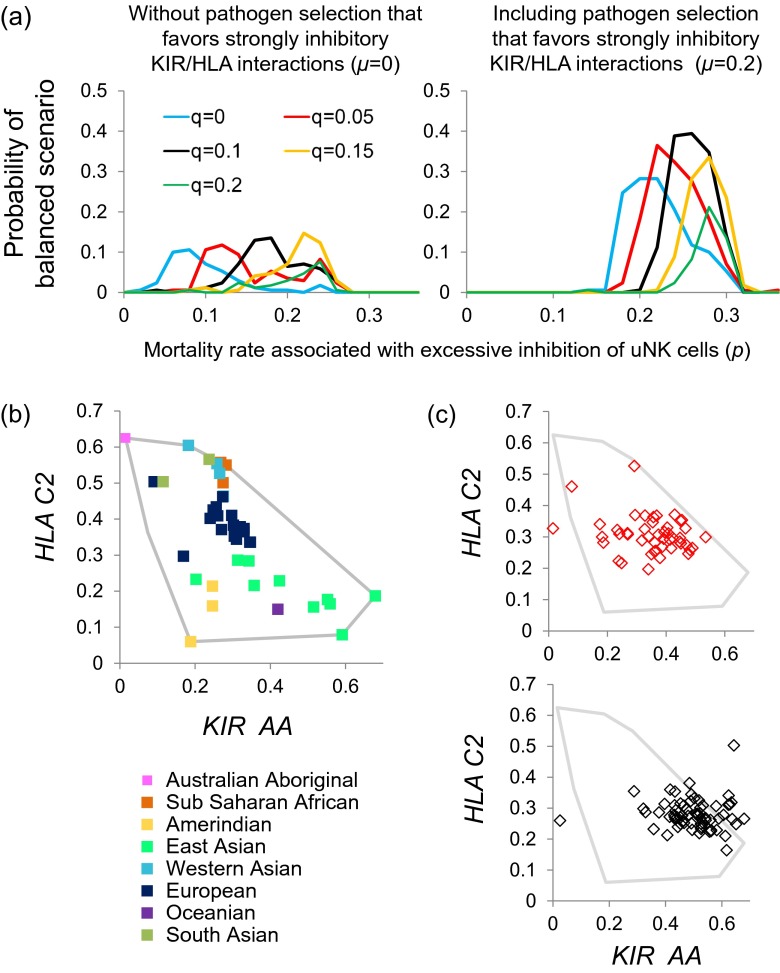
**Parameter combinations giving rise to a balance between** ***KIR A*** **and** ***KIR B***. We defined a balanced scenario as one in which the frequencies of *KIR A*-like and *KIR B*-like haplotypes (see key in Fig. 2a) were both ≥0.1, and the frequency of *HLA-C2* was >0.05 but <0.95. Panel **a** illustrates the probability of obtaining this outcome for different levels of mortality associated with excessive inhibition of uNK cells (*p*, *x*-axis); different levels of mortality associated with excessive activation of uNK cells (*q*, line colours), with or without a cost associated with a pathogen for which the level of NK cell activation/inhibition was important (*μ*, graph titles). Other parameters, the founder haplotype and threshold values were as given in the legend to Fig. 2. One hundred and seventy simulations were carried out at each combination of parameter values. Panel **b** illustrates the frequencies of *HLA-C2* and *KIR A* homozygosity observed globally. Representative population frequencies were taken from allelefrequencies.net (Gonzàlez-Galarza et al. 2015), see also ESM Methods. Panel **c** illustrates the frequencies of *HLA-C2* and *KIR A* homozygosity obtained in individual simulations that were classified as balanced. For all of panel **c**, *p* = 0.26 and *μ* = 0.2. The value of *q* is indicated by the colour of the markers in each graph.

We found that a balanced scenario (such as illustrated in Fig. 2), where *KIR A*-like and *KIR B*-like haplotypes and C1 and C2 are all present after 15,000 generations, was impossible to obtain without assuming some degree of selection from HLA-evading pathogens (Fig. S2, parameter *θ*). Without such selection, either C1 or C2 was likely to be lost from the population over the thousands of generations simulated, and without a diversity of HLA ligands there was no reason to maintain a specific KIR haplotypic structure. So long as selection from HLA-evading pathogens was present (*θ >* 0.1), the inclusion of at least a low level of reproductive selection whereby excessive inhibition of uNK cells can cause mortality, made a balanced scenario possible (Fig. S2 and Fig. 3a, first graph).

### Selection from pathogens for which strongly inhibited NK cells are advantageous increases the probability of obtaining a balance between *KIR A* and *KIR B*

We found that scenarios where *KIR A* and *KIR B* coexisted were obtained most frequently when we included not only reproductive selection and selection from HLA-evading pathogens, but also selection from pathogens for which strongly inhibited NK cells were particularly advantageous (Fig. 3a, second graph). Without selection in favour of strongly inhibited NK cells, the highest probability of the balanced scenario for any single set of parameters in Fig. 3 was 14.7 %; including such selection, the highest probability of obtaining a balanced scenario rose to 39.4 %. If strongly inhibitory KIRs are advantageous against certain pathogens, and therefore maintained in the population, certain pregnancies will experience excessive inhibition of NK cells, and suffer associated mortalities (i.e. eclampsia). This in turn generates a selection pressure for haplotypes containing activating KIRs, and makes the evolution of *KIR A* and *KIR B* more likely.

Figures 3b and c compare the frequencies of *KIR AA* genotypes and *HLA-C2* observed in human populations worldwide with the model’s output for two sets of parameters where a balanced scenario is likely. Although neither of the parameter combinations shown produce a negative correlation as strong as that observed in human data, our model can certainly produce frequency combinations within a plausible range.

### Different types of infectious disease selection cannot substitute for the combination of infectious disease selection and reproductive selection

**Fig. 4:**
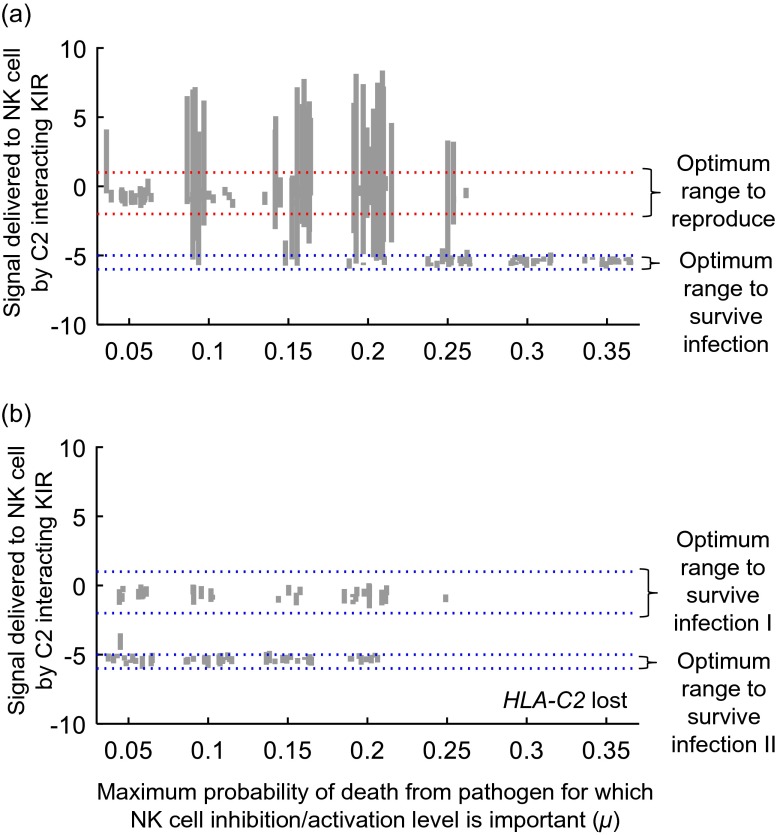
**Range of inhibition/activation signals delivered by C2 interacting KIRs after 15,000 generations of evolution under different conditions. **In our model we classify the KIRs encoded by each simulated haplotype both by their inhibitory or activating properties, but also by the magnitude of the signal delivered to the NK cell. In this figure, we classify all possible C2 interacting KIRs on a scale from highly inhibitory (large negative values) to highly activating (large positive values). The vertical grey lines indicate the interquartile range of all C2 interacting KIRs encoded by the haplotypes present after 15,000 generations of evolution in a given simulation. In panel **a**, reproductive selection, missing-self pathogen selection and pathogen selection that favors strongly inhibitory KIR/HLA interactions were all applied within each simulation (parameter values as given in the legend to Fig. 2). In panel **b** reproductive selection is not applied (*p*=0 and *q*=0, otherwise all parameter values as given in the legend to Fig. 2), and in addition to missing-self pathogen selection there were two independent challenges from pathogens for which the level of NK cell activation/inhibition was important. *x*_*L*_= -6 and *x*_*H*_= -5 for the first pathogen, and  *x*_*L*_= -2 and  *x*_*H*_= 1 for the second pathogen. Twenty simulations were carried out at each parameter combination, but grey lines are only shown if C2 was still present in the population after 15,000 generations. The costs associated with pathogens for which the level of NK cell activation/inhibition was important were varied as indicated on the x axis. At the start of each of these simulations, all individuals in the population were assigned *KIR* haplotypes generated as follows: each haplotype encoded a functional C1 interacting inhibitory KIR with a signal magnitude of 1.5; a functional C2 interacting inhibitory KIR with a signal magnitude randomly chosen from 5, 4 or 3, and a pseudogene C2 interacting inhibitory KIR with a signal magnitude randomly chosen from 5, 4 or 3.

Figure 4 illustrates the contrasting selective forces acting within our model in greater detail. Each of the vertical grey lines in the figure illustrates the range of inhibitory/activating behaviours exhibited by functional C2 interacting KIRs in a single simulated population after 15,000 generations of evolution. As shown in panel 4a, a combination of selection from reproduction and infectious disease encourages wider ranges of KIR-C2 interactions, from the inhibitory (negative values on the *y*-axis) to the activating (positive values on the *y*-axis) to coexist. It is in the regions of parameter space which allow such a wide range of KIR-C2 interactions that *KIR A* and *KIR B* haplotypes can emerge. If selection from infectious disease becomes too high (the far right hand side of panel 4a), the range of evolved KIR-C2 interactions narrows to match that which is best adapted to survive infection.

In panel 4b, we have applied no reproductive costs, but each individual in the population must survive challenge with pathogens for which strongly inhibited NK cells are optimal, and pathogens for which the optimal range of NK cell inhibition/activation is the same as that previously optimal for reproduction. Each simulation ends up with a set of possible KIR-C2 interactions that match one or other of the optimum ranges but we see none with a range of KIR-C2 interactions spanning both. This result suggests that it is difficult to create a balance between *KIR A* and *KIR B* using infectious disease selection alone—but there are many other factors, such as variable selection from different types of pathogens at different stages in human history, that we have not yet considered in our framework.

## Discussion

Specific examples of trade-offs between the capacity to either survive in the long term or maximise reproductive success in the short term are rare; although one excellent recent example is a negative association between antibody responsiveness and reproductive success observed in Soay sheep (Graham et al. 2010). A growing collection of studies suggests that *KIR* genes may be pivotal to a reproductive/immunological trade-off in humans (Hiby et al. 2014; Khakoo and Jamil 2011; Parham and Moffett 2013). The results presented here strongly suggest that present-day population genetic patterns exhibited by *KIR* haplotypes do indeed result from competing pressures to survive infection or to reproduce successfully.

Our model identifies a combination of reproductive selection and selection from HLA-evading pathogens as critical in allowing *KIR A* and *B* to emerge, but also shows that including an infection survival advantage for individuals with strongly inhibited NK cells makes a balance between *A* and *B*, alongside *HLA* alleles encoding C1 and C2 ligands, more likely. An infection advantage associated with strongly inhibited NK cells may follow from a process of ‘education’ or ‘licencing’ during NK cell development: specifically if stronger inhibitory signals during NK cell development set up the capacity for greater activation of NK cells during infection, as has been proposed for certain HLA-B/KIR3DL1 combinations in HIV patients (Martin et al. 2007). Alternatively, relatively strong inhibition of NK cells may help avoid immunopathologies associated with an over-reactive immune response. A recent observation in keeping with this hypothesis is that the relatively weakly inhibitory combination of KIR2DL3 and C1 is a risk factor for cerebral malaria, and the activating combination of KIR2DS1 and C2 may be a risk factor for non-cerebral severe malaria (Hirayasu et al. 2012). No infectious disease has yet been reported for which the specific combination of alleles encoding highly inhibitory KIR2DL1 receptors and their C2 ligands is of particular benefit. However, given the importance of such an effect in driving the evolution of *KIR A* and *KIR B* within our model, we predict that such a relationship should exist for at least some of the infectious diseases with which humans have coexisted for thousands of generations: e.g. malaria or similar pathogens which cause mortality prior to reproduction and are able to persist in non-urban social groups.

It is important to note that our model does not require that the best response to *all* pathogens involves strongly inhibited NK cells. It has been shown that *KIR B* is associated with higher IFN-γ release from NK cells upon stimulation with mycobacteria in vitro (Portevin et al. 2012), and multiple studies of *KIR* genes in tuberculosis patients suggest that greater NK cell inhibition is a risk factor for the disease (Braun et al. 2013; Mahfouz et al. 2011; Méndez et al. 2006; Shahsavar et al. 2012) [although not every tuberculosis study has found an effect (Tajik et al. 2012)]. It seems plausible that for some diseases, a swift inflammatory response that eliminates infection is best, whereas for other infections, the costs to the host of immunopathological damage are too high, and a less inflammatory response that nonetheless keeps the pathogen under control is preferable.

Our model assumes that when responding to any specific challenge, an individual with a narrow range of possible NK cell behaviours that is entirely optimised for that challenge will do better than an individual with a wider range of possible NK cell behaviours. However, the individual with narrow NK cell responses will, of course, perform badly against any challenge that requires NK cell responses outside of its range. Different ranges of optimum NK cell behaviours could derive from selection to survive infection and selection to reproduce, or could derive from infectious disease selection alone, if—as we have just noted—different ranges of NK cells responses are appropriate to different pathogens.

In Fig. 4, we explored whether *KIR A* and *B* haplotypes had to derive from a trade-off between surviving infection and reproducing or could be driven by competing pathogen selection pressures. Our results so far suggest that reproductive selection *per se* is important in the emergence of *KIR A* and *B*. In the presence of pathogen selection alone, a population is more likely to evolve a strategy where all genotypes are equally good at responding to one type of pathogen challenge, even if that means being equally poor at responding to others (Fig. 4b). It may be that the re-association of different HLA-C genotypes with the mother’s existing KIR genotype that is an integral part of the reproductive process is vital to encourage the persistence of diverse *KIR* haplotypes. This reassortment means that a *KIR* which happens to end up in a genotype for which the HLA-C ligands present made it relatively unfit, will later have the chance to encounter HLA-C ligands that render it relatively fit (or vice versa). Such a process seems inherently likely to encourage greater *KIR* diversity—and on one level the persistence of *KIR A* and *B* haplotypes in all human populations can be thought of as the persistence of a balance between diverse (activating and inhibitory) KIRs in all human populations.

We have focused on the effect of the KIR/ HLA interaction on NK cells. KIRs are, however, also expressed on a subset of T cells. It has been shown that the presence of KIR2DL2 tends to magnify either protective or detrimental effects of certain HLA alleles against viral infection (Seich al Basatena et al. 2011). The HLA alleles in question are not ones with which KIR2DL2 is likely to interact directly, and the authors suggest that the mechanism underlying their observations may involve KIR2DL2 increasing the lifespan of certain memory T cells. If T cells restricted by either advantageous or disadvantageous HLAs survive for longer in patients, their respective effects should both tend to be enhanced. Other inhibitory KIRs may have similar effects on the survival of T cells, and future models including more diverse HLAs could investigate the evolutionary significance of such a mechanism.

Recent computational studies have demonstrated that viral expression of decoy HLA class I molecules promotes KIR allelic diversity, and a combination of both activating and inhibitory KIRs should offer the maximum level of protection against such viruses (Carrillo-Bustamante et al. 2013, 2014). It seems likely, therefore, that selection from pathogens is responsible for the evolution of multiple KIRs, and multiple KIR/HLA interactions, in the first instance. However, the human-specific organisation of the *KIR* locus into *A* and *B* haplotypes calls for further explanation. As we have illustrated here, such organisation may well reflect selection from both infectious disease and reproductive processes.

## Electronic supplementary material

Below is the link to the electronic supplementary material.

ESM 1 (PDF 909 kb)
