# Supplementary material for: Reproduction, infection and killer-cell immunoglobulin-like receptor haplotype evolution
Source: Immunogenetics. 2016 Aug 12;68(10):755–64. doi: 10.1007/s00251-016-0935-9 (PMC5056949; doi:10.1007/s00251-016-0935-9)

Supplementary Material

Title: Reproduction, infection and Killer-cell Immunoglobulin-like Receptor haplotype evolution

Authors: Bridget S Penman[1*], Ashley Moffett[2], Olympe Chazara[2], Sunetra Gupta[1], Peter Parham[3,4]

Affiliations:

1. Department of Zoology, University of Oxford, UK OX13PS;

2. Department of Pathology, University of Cambridge, UK, CB2 1QP;

3. Department of Structural Biology, Stanford University, Stanford, CA 94035;

4. Department of Microbiology and Immunology, Stanford University, Stanford, CA 94035

*Corresponding author contact details: Department of Zoology University of Oxford, Tinbergen Building, South Parks Road, Oxford, UK, OX13PS; bridget.penman@zoo.ox.ac.uk; +44(0)1865 271132

Contents:

This supplementary material consists of (I) Supplementary Methods, (II) Supplementary Results (III) Supplementary Figures.

I. Supplementary Methods

The generation of NK cell activation state distributions from genotypes

KIRs do not operate in a vacuum – other receptors also provide stimulatory or inhibitory signals to NK cells. However, for the purposes of this study we are especially interested in the phenotypic consequences of possessing particular KIR/HLA-C ligand combinations. We thus define NK cell phenotypes in the following terms: what sorts of behaviours are an individual's NK cells capable of with respect to HLA-C*?*

As detailed in the main text, we defined each *KIR* allele within the model using the following 3 criteria: functional/non functional; encoding an activating or inhibitory receptor, and the magnitude of the signal its product could transmit to NK cells. We represented the variety of NK cell behaviours possible in an individual as a normal distribution of potential signals derived from KIR/HLA-C interactions, where the mean and standard deviation of that distribution were equal to the mean and standard deviation of the available KIR/HLA-C signals in an individual's genotype. Available signals were those deriving from functional KIRs present in the individual's genome, whose HLA ligands were also encoded in that individual's genome. In order to combine both activating and inhibitory KIRs in a single distribution, inhibitory KIRs contributed negative values and activating KIRs contributed positive values to the pool of available signals. This has the overall effect that, at the phenotypic level, an activating KIR can partially cancel out the effects of an inhibitory KIR, and vice versa.

In cases where only one KIR signal was possible (hence the standard deviation of the signals = 0), we set the standard deviation to 0.1, to generate a very narrow distribution around the single possible NK cell activation state. In the extremely unlikely but technically possible case of a genotype arising which contained no KIRs at all (either owing to one of the mutational processes included in the model having "switched" all the KIRs on a haplotype into non-functional pseudogenes, or owing to a separate mutational process in the

model having given all the KIRs in a haplotype a signal magnitude of zero, *and* such a haplotype subsequently being homozyogously inherited), that individual was assumed to be inviable.

In the case of pregnancy, distributions of possible uterine NK cell activation states were generated based on (i) the mother's *KIR* genotype and (ii) the fetal HLA ligand contributed by the father. Using only paternal HLA is a simplification, but it is in keeping with Hiby *et al*'s observations that it is paternally-derived C2 which increases the risk of pre-eclampsia and has the biggest effect on determining birth weight (Hiby *et al* 2004, 2010, 2014). Uterine NK cell activation state distributions were generated exactly as described in the preceding paragraphs, but as though for a *HLA-C2* homozygote if the paternal ligand happened to be C2, or as though for a *HLA-C1* homozygote if the paternal ligand happened to be C1.

Although our model is, necessarily, a simplified abstraction of reality, the following observations help to justify conceiving of NK cell behaviour on a spectrum, wherein more activating or more inhibitory KIRs may be able to partially cancel each other out at the phenotypic level:

(i) The risk of pre-eclampsia, which, as discussed in the main text, may be associated with excessively inhibited uterine NK cells, is diminished by the presence of *KIR* genes encoding activating receptors in the mother's genome. The reduction in risk appears to be proportional to the number of activating KIRs present (Hiby *et al* 2004).

(ii) Functional studies of NK cell behaviour have demonstrated that, in HLA-C2 homozygous donors, the expression of KIRDS1 on NK cells otherwise lacking KIRs (or on NK cells expressing KIR2DL3), makes those NK cells less responsive to stimulation by HLA negative target cells. However, the expression of KIR2DS1 does not render KIR2DL1-expressing cells from HLA-C2 homozygous donors less responsive to such stimulation (Fauriat *et al* 2010).

These observations are consistent with the notion that inhibitory KIRs can modulate the effects of activating KIRs and vice versa.

The signals derived from KIR/HLA interactions in our model are on an arbitrary scale, and hence have no unit. Their magnitudes only become meaningful in the context of the different thresholds set for the risks of death from infection or poor reproductive outcomes (see main text). By way of example, if the range of optimum NK cell activation states to survive infection ($x_L < x < x_H$) is defined as being between -5 and -2, then an inhibitory KIR of magnitude 4, which is scored in our model as a contribution of "-4" to the pool of available KIR signals in an individual's genome, is likely to be advantageous against infection because its presence may increase the proportion of the distribution of that individual's NK cell behaviours which fall between -5 and -2.

List of all events that occur within each generation of the simulation

- Every member of the population is challenged with (i) one or more different pathogens for which NK cell activation/inhibition state is important, and (ii) an HLA evading pathogen. Any deaths are recorded.
- Members of the population who died from one of the pathogen challenges are removed.
- *K* pregnancies are simulated. For each pregnancy, a maternal and paternal genotype are randomly sampled from the population. A fetal genotype is generated from the maternal and paternal genotypes by Mendelian inheritance. *HLA* and *KIR* genes are assumed to be completely unlinked, as is the case in the human genome. A distribution of uterine NK cell phenotypes is calculated based on potential interactions between maternal KIR and fetal HLA. This distribution is used to calculate a probability of (i) excessive inhibition of uNK cells leading to the death of both mother and infant or (ii) excessive activation of uNK cells leading to the death of both mother and infant. If the pregnancy escapes both of these outcomes, the new

offspring genotype forms part of the next generation. If the mother in a given pregnancy is deemed to have died, that maternal genotype is removed from the pool of potential parents before any subsequent pregnancies are generated.

Sources for *KIR AA* genotype and *HLA-C2* frequency data used in figure 3

KIR AA genotype and *HLA-C2* frequencies for a range of representative populations worldwide were obtained from the control population used in a study of pre-eclampsia in Uganda (Nakimuli *et al* 2015), and from the allelefrequencies.net online repository (Gonzàlez-Galarza *et al* 2015). In most cases, KIR and HLA frequencies were not obtained for exactly the same individuals in a single study – therefore we have matched separate studies on populations by ethnicity and geographical location. Specific allelefrequencies.net sources are detailed here. Each source is only listed once, although some sources provided information for more than one population.

<u>Australian aboriginal populations</u>

Toneva, M. *et al* (2001) Genomic diversity of natural killer cell receptor genes in three populations *Tissue Antigens* 57(4):358-362; *Major Histocompatibility Complex* (2000) Springer, Tokyo, edited by Masanori Kasahara; The 12th International Histocompatibility Workshop.

<u>Sub Saharan African populations</u>

Norman, P. *et al*. (2013) Co-evolution of Human Leukocyte Antigen (HLA) Class I Ligands with Killer-Cell Immunoglobulin-Like Receptors (KIR) in a Genetically Diverse Population of Sub-Saharan Africans *PLoS Genet* 9(10): e1003938; Du, Z. *et al* (2007) Receptor-ligand analyses determine minimal killer cell Ig-like receptor (KIR) in humans *Immunogenetics* 59(1): 1-15; Unpublished data uploaded to allelefrequencies.net with the following identifiers: C Navarette. L Creary_2008 and M.Fernandez-Vina_2001.

<u>Amerindian populations</u>

Proceedings of the 13th International Histocompatibility Workshop (2006); García-Ortiz, J.E. *et al* (2006) High-resolution molecular characterization of the HLA class I and class II in the Tarahumara Amerindian population *Tissue Antigens* 68(2):135-46; Layrisse, Z *et al* (2001)

Extended HLA haplotypes in a Carib Amerindian population: the Yucpa of the Perija Range *Hum Immunol* 62(9):992-1000; Gutiérrez-Rodríguez, M.E. *et al* (2006) KIR Gene in Ethnic and Mestizo Populations from Mexico *Human Immunology* Volume 67 (1-2): 85-93; Norman, P. *et al* (2007) Unusual selection on the KIR3DL1/S1 natural killer cell receptor in Africans *Nature Genetics* 39: 1092-1099; Gendzekhadze, K. *et al* (2006) High KIR diversity in Amerindians is maintained using few gene-content haplotypes *Immunogenetics* 58(5-6):474-80.

<u>West Asian populations</u>

Buhler, S. *et al* (2002) PCR-SSOP molecular typing of HLA-C alleles in an Iranian population *Tissue Antigens* 59: 525-30; Buhler, S. *et al* (2006) HLA-C molecular characterization of a Lebanese population and genetic structure of 39 populations from Europe to India–Pakistan *Tissue Antigens* 68: 44-57; Tajik, N *et al* (2009) Distribution of KIR genes in the Iranian population *Tissue Antigens* 74(1): 22–31; Hajeer’ A.H. *et al* (2013) HLA-A, -B, -C, -DRB1 and -DQB1 allele and haplotype frequencies in Saudis using next generation sequencing technique. *Tissue Antigens* 82(4) 252–258; Unpublished data uploaded to allelefrequencies.net with the following identifiers: R. Mahfouz_2004, Awad E. Osman_2012.

<u>South Asian populations</u>

Shankarkumar, U. *et al* (2001) HLA antigen distribution in Maratha community from Mumbai, Maharastra, India *Inter J Human Genet* 1(3): 173–7; Kulkarni, S. et al (2008) Comparison of the rapidly evolving KIR locus in Parsis and natives of India *Immunogenetics* 60(3-4):121-9; Mohyuddin, A. and Mehdi, S.Q (2005) HLA analysis of the Parsi (Zoroastrian) population in Pakistan *Tissue Antigens* 66(6): 691-695; Norman, P. (2002) Natural killer cell immunoglobulin-like receptor (KIR) locus profiles in African and South Asian populations *Genes and Immunity* 3 86–95.

East Asian populations

Chen S *et al* (2006) Allelic distribution of HLA class I genes in the Tibetan ethnic population of China *Int J Immunogenet* 33(6):439-45; Zhu, B. *et al* (2010) Killer cell immunoglobulin-like receptor gene diversity in the Tibetan ethnic minority group of China *Human Immunology* 71(11): 1116–1123; Yao, Y. *et al* (2009) Distribution of HLA-A, -B, -Cw, and -DRB1 alleles and haplotypes in an isolated Han population in Southwest China *Tissue Antigens* 73 (6) 561–568; Shi, L. *et al* (2011) Distribution of killer cell immunoglobulin-like receptor genes and combinations with HLA-C ligands in an isolated Han population in southwest China *Tissue Antigens* 78(1) 60–64; Chen, S. *et al* (2007) Origin of Tibeto-Burman speakers: Evidence from HLA allele distribution in Lisu and Nu inhabiting Yunnan of China Human *Immunology* 68(6) 550–559; Yao, Y. *et al* (2011) Diversity of killer cell immunoglobulin-like receptor genes in four ethnic groups in China *Immunogenetics* 63(8) 475-483; Shi, L. *et al* (2010) Genetic link among Hani, Bulang and other Southeast Asian populations: evidence from HLA -A, -B, -C, -DRB1 genes and haplotypes *distribution International Journal of Immunogenetics* 37(6) 467–475 ; Itoh, Y. *et al* (2005) High-throughput DNA typing of HLA-A, -B, -C, and -DRB1 loci by a PCR–SSOP–Luminex method in the Japanese population *Immunogenetics* 57: 717–729; Yawata, M. *et al* (2006) Roles for HLA and KIR polymorphisms in natural killer cell repertoire selection and modulation of effector function *Journal of Experimental Medicine* 203 (3): 633; Proceedings of the 13th International Histocompatibility Workshop (2006); Chuan Lee, Y. *et al* (2008) Asian population frequencies and haplotype distribution of killer cell immunoglobulin-like receptor (*KIR*) genes among Chinese, Malay, and Indian in Singapore *Immunogenetics* 60(11): 645-654; Whang, D.H. *et al* (2005) Haplotype analysis of killer cell immunoglobulin-like receptor genes in 77 Korean families *Human Immunology* 66(2) 146-154; Yang, K-L. *et al* (2009) High-resolution human leukocyte antigen (HLA) haplotypes and linkage disequilibrium of HLA-B and -C and HLA-DRB1 and -DQB1 alleles in a Taiwanese population *Human Immunology* 70(4) 269-276; Wu, G-Q. *et al* (2009) Distribution of killer-cell immunoglobulin-like receptor genes in

Eastern mainland Chinese Han and Taiwanese Han populations *Tissue Antigens* 74(6): 499-507; The 7th Asia-Oceania Histocompatibility Workshop and Conference ; Chaisri, S. *et al* (2013) Polymorphisms of killer immunoglobulin-like receptors (KIRs) and HLA ligands in northeastern Thais. *Immunogenetics* 65(9):645-53; Hoa, B.K. *et al* (2008) HLA-A, -B, -C, -DRB1 and -DQB1 alleles and haplotypes in the Kinh population in Vietnam. *Tissue Antigens*. 71(2):127-34; Unpublished data uploaded to allelefrequencies.net with the following identifier: Chul-Woo Pyo, Seong-Suk Hur, Yang-Kyum Kim, Hyong-Jae Kim, Ji-Yeon Choi, Hee-Baeg Choi, Tai-Gyu Kim_2000.

European populations

HLA 1998 edited by Paul Terasaki and David Gjertson (page 119); Proceedings of the 13th International Histocompatibility Workshop (2006); Pavlova, Y. *et al* (2008) Distribution of KIR genes in the Czech population *International Journal of Immunogenetics* 35 (1): 57–61; Alizadeh, M. *et al* 2008 Evidence for the improvement of French Volunteer Bone Marrow Donor Registry by using HLA-SBT at the registration step (abstract for poster presentation) *Tissue Antigens* 71(4): 315; Denis, L. *et al* (2005) Genetic diversity of KIR natural killer cell markers in populations from France, Guadeloupe, Finland, Senegal and Réunion *Tissue Antigens* 66(4): 267–276; Schmidt, A.H. *et al* (2009) Estimation of high-resolution HLA-A, -B, -C, -DRB1 allele and haplotype frequencies based on 8862 German stem cell donors and implications for strategic donor registry planning *Hum Immunol* 70(11):895-902; Becker, S. *et al* (2003) Assessment of killer cell immunoglobulin like receptor expression and corresponding HLA class I phenotypes demonstrates heterogenous KIR expression independent of anticipated HLA class I ligands *Human Immunology* 64(2): 183–193; Williams, F. *et al* (2002) Molecular diversity of the HLA-C gene identified in a Caucasian population *Human Immunology* 63(7) 602-613; Guerini, F.R. *et al (2008)* HLA-Cw allele frequencies in northern and southern Italy Transplant Immunology 18(3): 286–289; Harbo, H.F. *et al* (2010) Norwegian Sami differs significantly from other Norwegians according to their HLA profile Tissue Antigens 75(3): 207-217; Karlsen, T.H. *et al* (2007) Particular

genetic variants of ligands for natural killer cell receptors may contribute to the HLA associated risk of primary sclerosing cholangitis *Journal of Hepatology* 46(5): 899–906; Nowak, I. *et al* (2010) Does the KIR2DS5 Gene Protect from Some Human Diseases? *PLoS One* 5 e12381; Bubnova ,L. Poster presentation P28 EFI 2012; Comas, D. *et al* (1998) HLA class I and class II DNA typing and the origin of Basques. *Tissue Antigens* 51(1):30-40; Santin, I. *et al* (2006) Killer Cell Immunoglobulin-Like Receptor (*KIR*) Genes in the Basque Population: Association Study of *KIR* Gene Contents With Type 1 Diabetes Mellitus *Human Immunology* 67(1-2): 118-124; Muro, M. *et al* (2001) HLA polymorphism in the Murcia population (Spain): in the cradle of the archaeologic Iberians *Human Immunology* 62(9):910-921; Campillo JA *et al* (2006) HLA class I and class II frequencies in patients with cutaneous malignant melanoma from southeastern Spain: the role of HLA-C in disease prognosis. *Immunogenetics* 57(12):926-33; Middleton, D. *et al* (2007) No association of *KIR* genes with Behcet's disease *Tissue Antigens* 70(5), 435–438; Bunce, M. et al (1997) High resolution HLA-C typing by PCR-SSP: identification of allelic frequencies and linkage disequilibria in 604 unrelated random UK Caucasoids and a comparison with serology *Tissue Antigens* 50(1): 100-111; Mack, S.J. *et al* (2009) HLA-A, -B, -C, and -DRB1 allele and haplotype frequencies distinguish Eastern European Americans from the general European American population *Tissue Antigens* 73(1): 17–32; Hollenbach J. A. *et al* (2010) Report from the killer immunoglobulin-like receptor (KIR) anthropology component of the 15th International Histocompatibility Workshop: worldwide variation in the KIR loci and further evidence for the co-evolution of KIR and HLA *Tissue Antigens* 76(1): 9–17; Unpublished data uploaded to allelefrequencies.net with the following identifiers: Cintia Yanina Marcos_2005; L.Zahlavova,N.Bendukidze,E.Ivaskova_2002; Dr. Christian Demanet Sonja Verheyden_2001; J Sivula, J Partanen_2004; D. Middleton _2003; M Martinetti & A Pasi_2007; Jacek Nowak, Renata Mika-Witkowska, Marta Rogatko-Koros, Joanna Dziopa_2013; Mats Bengtsson_2013; Marie Schaffer _2011; Guher Saruhan Direskeneli _1997; D Harvey_2006.

Oceanian populations

Proceedings of the 13th International Histocompatibility Workshop (2006); Velickovic, M. et al (2006) Diversity of killer cell immunoglobulin-like receptor genes in Pacific Islands populations *Immunogenetics*.58(7):523-32

II. Supplementary Results

To investigate the sensitivity of our results to the specific mutation rates used in each simulation, we applied the combination of reproductive and infectious disease selection used in figure 2 of the main text, and varied mutation rates m_1, m_2 and m_3 and the recombination rate (r). As illustrated in figure S1, it was possible to obtain balanced outcomes with a range of values for each, so the results presented in the main text are not reliant on the specific mutation rates chosen.

Nevertheless, some patterns do appear in figure S1. It seems that a balanced outcome is only very rarely obtained if the mutation rate m_3 is lower than 10^{-7}. m_3 is the rate at which inhibitory KIRs can become activating KIRs, or vice versa. In a population of size 1000 where $m_3 = 10^{-7}$, such a mutation would arise on average only once every 10,000 generations. Since we only simulated 15000 generations, and since our founder haplotype contained no activating KIRs, it is unsurprising that this restriction applies.

The fact that we can still obtain the balanced scenario in many cases when m_1, m_2 or r are less than or equal to 10^{-7} shows that these other mutational processes are, individually, less important to our overall result. However, panel (c) of figure S1 clearly demonstrates that when the probability of a change in the signal magnitude of each KIR is extremely low ($m_1 = 10^{-7}$), switching KIRs on and off (m_2) must also occur at a relatively high rate, unless the rate of switching between activating and inhibitory states (m_3) is extremely high.

III. Supplementary Figures

Figure S1: Sensitivity analysis of parameters m_1, m_2, m_3 and r. We let the values of m_2, m_3 and r be equal to 10^{z1}, 10^{z2} and 10^{z3}, where z1 and z2 were each drawn from a range between -3 and -8, and z3 was drawn from a range between -1 and -8, using Latin Hypercube Sampling. For each of three different values of m_1 (indicated in the title of each panel), 4000 simulations were carried out. The plots indicate combinations of m_2 (x axis); m_3 (y axis) and r (marker colour) which generated a balanced outcome (see legend to figure 3). The founder haplotype, parameter values other than m_1, m_2, m_3 and r, and threshold values were as given in the legend to figure 2, with the exception that p=0.25.

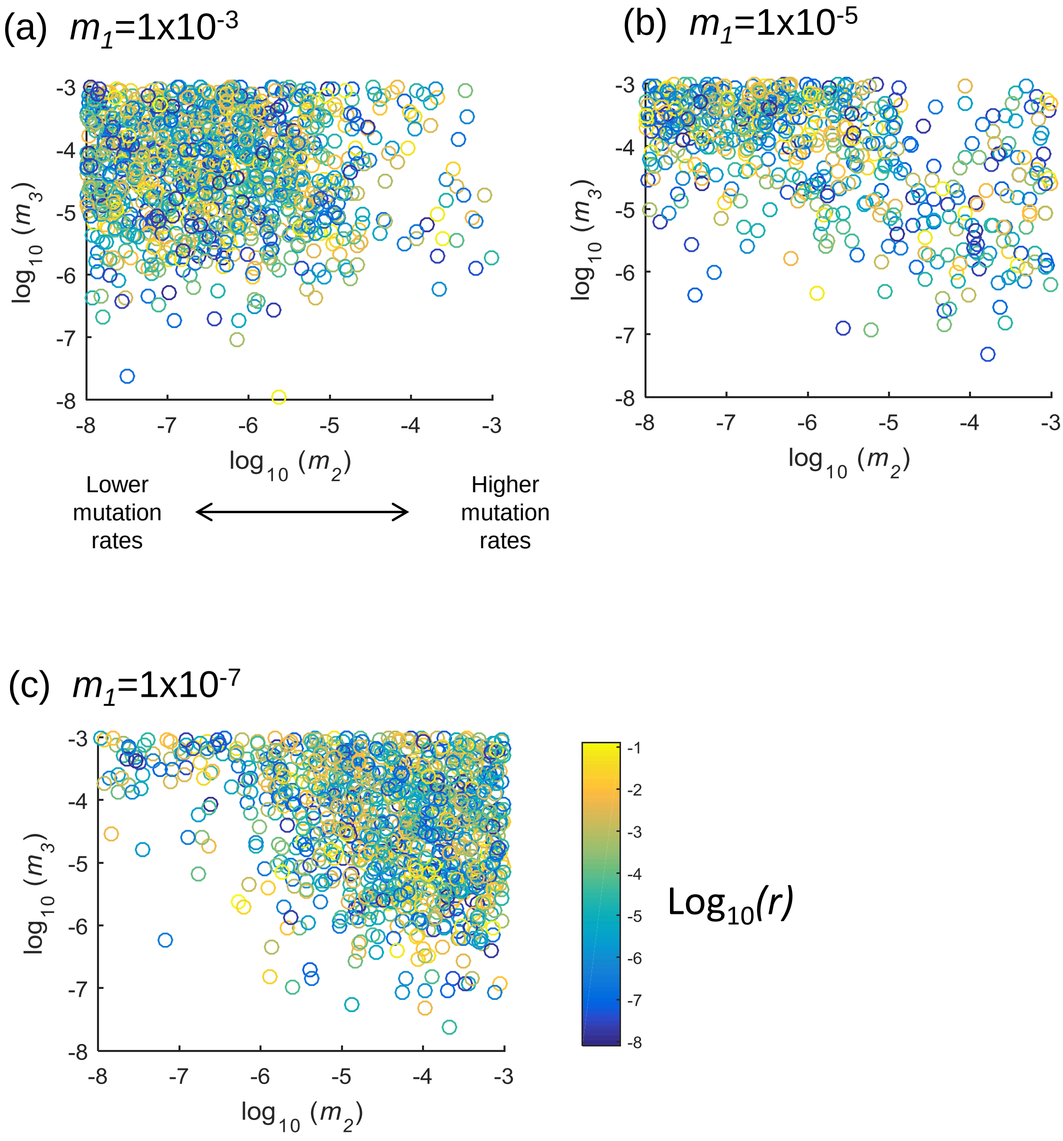

Figure S2: Sensitivity analysis of parameters *θ*, *μ*, *p* and *q*. We used Latin Hypercube Sampling where each of *θ*, *μ*, *p* and *q* could range between a minimum value of 0 and a maximum value of 0.4. 7500 simulations were carried out. The histograms in panel (a) illustrate the distribution of values of each parameter from the parameter combinations which were associated with a balanced outcome (see legend to figure 3). It seems particularly critical that both *p* and *θ* be nonzero if a balanced outcome is to arise. The scattergraph in panel (b) illustrates the location of the balanced scenarios within the whole parameter space. The founder haplotype, threshold values and all parameter values other than *θ*, *μ*, *p* and *q* were as given in the legend to figure 2.

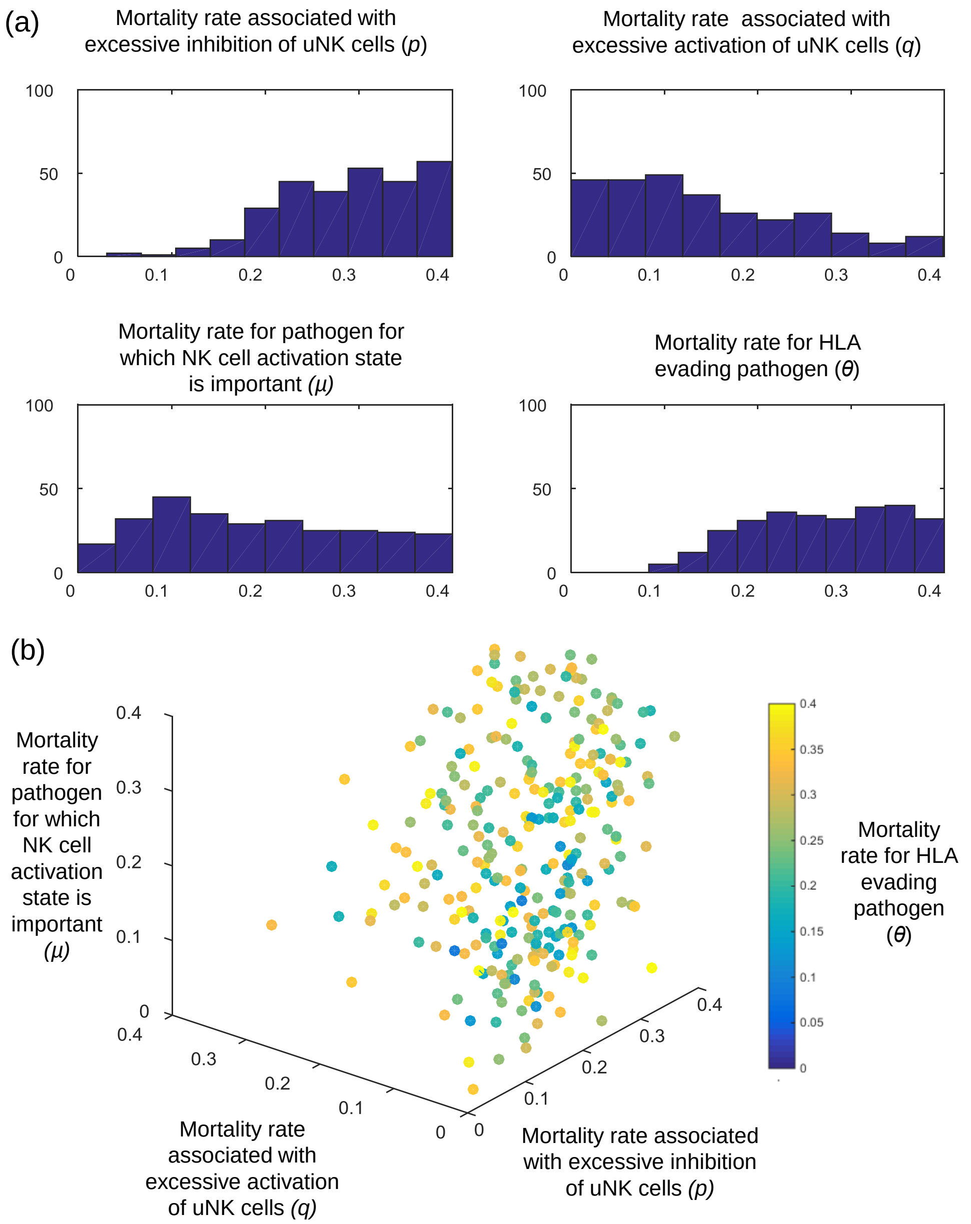

Supplement: Supplementary file 1 — (PDF 909 kb) [file 251_2016_935_MOESM1_ESM.pdf]
